# Vascular Cell Adhesion Molecule-1 (VCAM-1) contributes to macular fibrosis in neovascular age-related macular degeneration through modulating macrophage functions

**DOI:** 10.1186/s12979-023-00389-x

**Published:** 2023-11-20

**Authors:** Wen Deng, Caijiao Yi, Wei Pan, Jian Liu, Jinyan Qi, Juan Chen, Zengchao Zhou, Yiqin Duan, Xiangyan Ning, Jun Li, Changhua Ye, Zhongping Chen, Heping Xu

**Affiliations:** 1 Aier School of Ophthalmology, Central South University, Changsha, China; 2 Aier Institute of Optometry and Vision Science, Changsha, China; 3 Changsha Aier Eye Hospital, Changsha, China; 4 Wellcome-Wolfson Institute for Experimental Medicine, School of Medicine, Dentistry and Biomedical Sciences, Queen’s University Belfast, Belfast, BT9 7BL UK

**Keywords:** Macular fibrosis, Vascular cell adhesion molecule 1, Macrophage, Inflammation, Aqueous humour

## Abstract

**Background:**

Neovascular age-related macular degeneration (nAMD) is a major cause of blindness in the elderly. The disease is due to the growth of abnormal blood vessels into the macula, leading to the loss of central vision. Intravitreal injection of vascular endothelial growth factor (VEGF) inhibitors (e.g., anti-VEGF) is the standard of care for nAMD. However, nearly 50% of patients do not respond or respond poorly to the therapy. More importantly, up to 70% of nAMD patients develop macular fibrosis after 10 years of anti-VEGF therapy. The underlying mechanism of nAMD-mediated macular fibrosis is unknown although inflammation is known to play an important role in the development of abnormal macular blood vessels and its progression to fibro-vascular membrane. In this study, we measured the intraocular levels of adhesion molecule VCAM-1, ICAM-1, CD44, CD62L, and CD62P in nAMD patients with and without macular fibrosis and investigated the link between the levels of adhesion molecule and clinical features (e.g., visual improvement, retinal thickness, etc.). We further investigated the effect of VCAM-1 in macrophage function in vitro and the development of subretinal fibrosis in vivo using a two-stage laser-induced protocol.

**Results:**

The aqueous levels of ICAM-1, VCAM-1, CD44, and CD62L were significantly higher in nAMD patients compared to cataract controls. The aqueous level of VCAM-1 (but not other adhesion molecules) was significantly higher in patients with macular fibrosis than those without and the level correlated positively with the retinal thickness. VCAM-1 was highly expressed at the lesion site in the mouse model of subretinal fibrosis. Blocking VCAM-1 or its receptor VLA-4 significantly prevented macrophage infiltration and reduced subretinal fibrosis in vivo. VCAM-1 induced macrophage migration and upregulated the expression of *Arg-1, Mmp12* and *Il6* but down-regulated the expression of *iNOS* and *Il1b* in macrophages.

**Conclusions:**

VCAM-1 may contribute to the development of macular fibrosis in nAMD patients by modulating macrophage functions, including migration and profibrotic polarization.

**Supplementary Information:**

The online version contains supplementary material available at 10.1186/s12979-023-00389-x.

## Background

Neovascular age-related macular degeneration (nAMD) is the leading cause of blindness in the elderly in developed countries [1]. The disease is characterized by abnormal growth of blood vessels into the macula leading to the loss of central vision. There are different subtypes of nAMD, including choroidal neovascularization (CNV), polypoidal choroidal vasculopathy (PCV) and retinal angiomatous proliferation (RAP) [2, 3]. Intravitreal injection of vascular endothelial growth factor (VEGF) inhibitor is the standard of care for nAMD [4]. However, up to 15% ~ 50% of patients do not respond or respond poorly to the therapy [5–7], and 40 ~ 70% of treated eyes can develop macular fibrosis within 10 years [8]. The fibrotic scar destroys the neuronal macula leading to unreversible loss of visual function. The clinical challenges highlight the need for developing alternative and better therapies. To achieve this, an improved understanding of the disease pathogenesis is needed.

Risk factors of nAMD-mediated macular fibrosis include intraretinal fluids, haemorrhage, initial larger CNV lesion, delayed treatment, and old age [9, 10]. These risk factors highlight the role of sustained inflammation in converting the diseased blood vessels into fibrovascular membranes in nAMD. Inflammation is known to play an important role in the pathogenesis of AMD [11, 12]. We and others have shown that circulating immune cells from nAMD patients are more active [6, 13] and produce higher levels of inflammatory cytokines and chemokines (e.g., IL-8 and CCL2) [14] than those from age-matched healthy controls. Patients with macular fibrosis, in particular, have higher circulating levels of immune mediators such as neutrophil gelatinase-associated lipocalin (lipocalin-2) [15] and complement proteins [16]. Fibrovascular lesions from nAMD patients contain various immune cells including T cells and macrophages [17]. Macrophages are known to contribute critically to the development of retinal angiogenesis [18] and fibrosis [19, 20] in nAMD. Exactly how immune cells are recruited to the macular lesion in nAMD remains poorly defined.

Leukocyte trafficking in inflammation involves a cascade of events including tethering, rolling, firm adhesion and transendothelial migration. The recruitment of immune cells is governed by various chemokines and adhesion molecules. For example, CD44 is known to be critically involved in leukocyte trafficking in the inflamed retina [21], T cell migration across the blood-retinal barrier (BRB) is regulated by chemokine macrophage inflammatory protein-1α (MIP-1 α) [22] and P-selectin glycoprotein (CD62P) [23], whereas monocyte/macrophage trafficking at the BRB is controlled by chemokine CCL2 [24] and its receptor CCR2 [25] as well as adhesion molecules CD62L and CD44 [26]. In nAMD, adhesion molecules such as Intercellular Adhesion Molecule 1 (ICAM-1) and Vascular Cell Adhesion Molecule 1 (VCAM-1) have been detected in choroidal neovascularization (CNV) [27] and silencing VCAM-1 mRNA expression could suppress experimental CNV [28]. Higher intraocular levels of adhesion molecules have been observed in AMD patients [27, 29, 30]. However, their link to macular fibrosis remains elusive.

In this study, we investigated the aqueous levels of ICAM-1, VCAM-1, CD44, CD62L and CD62P in nAMD patients with and without macular fibrosis and found that VCAM-1 is significantly higher in patients with macular fibrosis. We further found that the VCAM-1 could induce macrophage migration and promote macrophage polarization towards an alternatively activated profibrotic phenotype. Importantly, we found that blocking VCAM-1 or its receptor VLA-4 significantly suppressed CNV-mediated subretinal fibrosis in a mouse model.

## Methods

### Study participants

The study protocol was approved by the Research Ethics Committee of Changsha Aier Eye Hospital (Ethical batch number: AIER2019IRB12) and was in accordance with the tenets of the Declaration of Helsinki on research into humans. Informed consent was obtained from all participants. Forty-eight nAMD and 24 senile cataract patients were recruited from Changsha Aier Eye Hospital during 2020.12.01 and 2022.03.22 to this study.

The inclusion criteria were (1) older than 55 years of age; (2) diagnosed with nAMD (including CNV and PCV) by medical retina specialists following fundus photography, optical coherence tomography angiography (OCTA), fluorescein fundus angiography (FFA), and indocyanine green angiography (ICGA). Exclusion criteria were (1) history of ocular trauma; (2) presence of other eye diseases such as pathological myopia, diabetic retinopathy, and glaucoma; (3) history of intraocular surgery or laser therapy within 6 months; (4) presence of intraocular or systemic inflammatory or autoimmune diseases; (5) the use of immunosuppressive medications such as steroids. The inclusion criteria of the control group were elderly cataract patients who needed phacoemulsification and the exclusion criteria were the same as above.

### Clinical examination

Comprehensive ophthalmic examinations were performed at baseline, including best corrected visual acuity (BCVA), intraocular pressure (IOP), axial length, slit lamp examination, fundus stereoscopic biomicroscopy, colour fundus photography, FFA, ICGA and OCTA. Data on axial length were collected for both the study group and control group to rule out myopia-associated CNV. All patients received intravitreal anti-VEGF (ranibizumab or conbercept) treatment at their first visit, followed by 3 × monthly injections and then subjected to a ‘injections as pro re nata’ (PRN) regime during monthly follow-up visits. Monthly follow-up examinations included BCVA, slit lamp-assisted fundus biomicroscopy and OCTA. The improvement of BCVA and the changes in central retinal thickness (CRT) were calculated by subtracting the values (LogMar score for BCVA, retinal thickness of CRT) collected from the time of the intravitreal anti-VEGF injection from the values collected at the next fellow up visit. The presence or absence of subretinal macular fibrosis was assessed by fundus examinations (colour photography, FFA and OCTA) at the first hospital visit.

### Aqueous sample collection

Aqueous humour from nAMD patients was collected during intravitreal injection of anti-VEGF. Briefly, anterior chamber paracentesis was performed to control the intraocular pressure, and ~ 60μL of aqueous humor was collected aseptically using a 1 ml disposable sterile syringe in an ophthalmology surgical operating room. Aqueous humor from cataract patients was collected during phacoemulsification surgery. The samples were placed into a sterile 1.5 mL Eppendorf tube and stored at − 80 °C freezer until analysis.

### Measurement of adhesion molecules

The aqueous humor samples were thawed on ice and centrifuged at 3000 rpm for 5 min. The levels of ICAM-1, VCAM-1, CD44, CD62L and CD62P were determined through a magnetic bead-based multiplex assay (Human Magnetic Luminex® Assay, R&D Systems Inc.) following the manufacturer's specifications. For each measurement of ICAM-1, VCAM-1, CD44, and CD62L, 50μL of supernatants (25μL for CD62P) were diluted to 100μL using sample diluent buffer immediately prior to the assay. In brief, the samples were incubated for 2 h with a specific cocktail of antibodies pre-coated onto magnetic microparticles, followed by 1 h incubation with biotinylated antibodies cocktail and additional 30 min incubation with streptavidin–phycoerythrin conjugate (Streptavidin-PE), which binds to the biotinylated antibody. Finally, the microparticles were resuspended in a buffer and read using a MagPix™ System (Bio-Rad Laboratories, Inc.). The data were calculated using the spline curve-fitting method (Milliplex Analyte, Application Version: 5.1.0.0).

### Animals

C57BL/6 J mice were purchased from the SJA Laboratory Animal Co., Ltd (Changsha, China).

Mice lived under a 12-h light/dark cycle in pathogenic-free conditions with open access to dry feed and water in the Department of Laboratory Animals of Hunan Normal University, China. All animal-related procedures were conducted following the Association for Research in Vision and Ophthalmology (ARVO) Statement for the Use of Animals in Ophthalmic and Vision Research and the protocols were approved by the Animal Welfare Ethics Committee of Hunan Normal University.

### Induction of subretinal fibrosis

Subretinal fibrosis was induced using a two-stage laser protocol detailed previously by us [31]. Briefly, mice were anaesthetized with an intraperitoneal injection of sodium pentobarbital (60 mg/kg, Sigma Aldrich), and pupils were dilated with 0.5% tropicamide and 0.5% phenylephrine (Santen Pharmaceutical, JPN). Each eye received four laser burns (Laser settings: 200 mW power, 100 ms duration and 60 μm spot size, Topcon, 532 nm Laser, JPN). Seven days later, a second laser burn was applied to each lesion using the same setting.

### Intravitreal injection

We conducted two treatments in mice with subretinal fibrosis: (1) anti-VCAM-1 antibody treatment and vehicle control (PBS), (2) VLA-4 inhibitor treatment and vehicle control (dimethyl sulfoxide, DMSO). 6–8 mice were included in each group. One microliter (1 µL) of anti-VCAM-1 antibody (6.85 µg, BE0027, BioXcell, endotoxin < 0.002EU/µg, Lebanon, USA) or VLA-4 inhibitor (8.17 µg, BIO5192, MedChemExpress LLC, USA) or relevant vehicle was administered via intravitreal injection immediately after the second laser. Mice were sacrificed 10 days after the second laser and eyes were collected and processed for further investigation.

### Immunofluorescence staining

Mouse eyes were fixed in 4% paraformaldehyde (PFA) for 4 h and processed for either cryosections or RPE/choroid flatmounts preparation. For cryosections, mouse eyes were embedded in optimal cutting temperature (OCT) and cryosectioned with 10 µm thickness. The sections were blocked with 10% goat serum and 2% BSA, permeabilized with 0.1% Triton X-100 for 1 h, followed by incubation with the cocktails of primary antibodies at 4 °C overnight. After thorough washes, samples were incubated with Alexa Fluor 594 or Alexa Fluor 488 conjugated secondary antibodies for 1.5 h at room temperature. The detailed information of the antibodies, including the sources, catalog numbers and dilutions are listed in Supplementary Table S1.

For RPE/choroid flatmount staining, after blocking and permeabilization, the samples were incubated with rabbit anti-collagen-1 and overnight, followed by incubation with Alexa Fluor 594-conjugated goat anti-rabbit IgG and Alexa Fluor 488-conjugated donkey anti-rat IgG. The samples were counter-stained with 4′,6-diamidino-2-phenylindole (DAPI, Cat: D8200, Solar-bio) to illustrate cell nuclei and imaged by using the Zeiss LSM 880 Confocal Microscope (Zeiss, Braunschweig, Germany).

### Quantitative real‑time PCR

Total RNAs were extracted from RPE-choroid or bone marrow-derived macrophages (BMDMs) using the miRNeasy Micro Kit (Cat: 1071023, Qiagen, Dusseldorf, Germany) or total RNA Kit II (Cat: R6934-01, Omega, Norcross, GA) respectively following manufacturer’s instructions. Then the extracted RNA was used to synthesize cDNA via the PrimeScript RT Reagent Kit (Cat: 6110A, Vazyme Biotech, Nanjing, China). Quantification of gene expression was performed by real-time PCR using SYBR green fluorescence (Cat: Q711-02, Vazyme Biotech) on a LightCycler 96 (Roche, Basel, Switzerland) (Primers are listed in Supplementary Table S2). Target gene expression was calculated using the ΔΔCt method and was normalized to *Gapdh* expression levels.

### Isolation and culture of bone marrow-derived macrophage

Murine bone marrow cells were isolated and cultured as previously described [32]. In brief, femurs and tibiae were isolated from mice, ensuring that joints were kept intact, and placed in cold DMEM (Cat: 11,995, Gibco) containing 1% penicillin/streptomycin (P/S). The muscle was removed using a sterile scalpel and scissors, the ends of the bones were cut in a ventilated hood, and bone marrow cells were flushed out with cold DMEM. Cells were filtered through a 40-μm cell strainer, centrifuged at 1100 rpm for 5 min, and the supernatant was then removed. Subsequently, the red blood cells were removed with lysis buffer and followed by centrifugation to elute the lysate. Cells were resuspended in DMEM medium containing 20% L929-conditioned medium, 15% FBS (Cat:BS-1102, OPCEL, Inner Mongolia Opcel Biotechnology Co., Ltd), 1% P/S. and seeded in 10 cm nontreated petri dish for 6–7 days to generate bone marrow derived-macrophages (BMDM). Differentiation was assessed using the flow cytometry and the associated antibodies anti-mouse F4/80-FITC (1:100, Cat: 11–4801-82, eBioscience) and anti-mouse CD11b-PerCP/Cy5.5 (1:100, Cat:101227, Biolegend) were stained for mature BMDM. Then the cells were collected for further study (see below).

### In vitro treatment of macrophages with VCAM-1

The differentiated mature BMDM were seeded in 6 well plates and treated with different concentrations (100, 500 and 1000 ng/ml) of recombinant murine VCAM-1 (Cat: 643-VM, R&D) for 24 h in DMEM without FBS. Then cells were thoroughly washed with PBS and harvested for RT-qPCR.

### Macrophage migration assay

Cell migration assays were performed in a 24-well plate Boyden chamber (8-µm pore size, Cat: 3422, Corning). Briefly, BMDMs were starved overnight before seeding into the insert (10^5^ cells/well). The recombinant murine VCAM-1 (25 µg/ml) in serum-free DMEM was added to the bottom chambers. After incubating at 37^◦^C and 5% CO_2_ for 10 h with or without anti-VCAM-1 neutralizing antibody (20 µg/ml) or VLA-4 inhibitor (2, 10 µM) in the inserts. The transwell inserts were removed and the cells were fixed with 4% paraformaldehyde in PBS for 10 min and processed for DAPI staining. The cells remaining on the inner side of the insert were removed with a cotton swab. An inverted fluorescence microscope (10 × objectives, Zeiss) was used to obtain images of the cells attached to the outer side of the insert membrane. Images from five random visual fields from each sample were used to count the cell numbers using Image J software (Version 2.9.0; Java 1.8.0).

### Statistical analysis

The clinical data were analysed using the Statistical Package for the Social Sciences (version 25.0; IBM SPSS Statistics, Armonk, NY, USA). Categorical demographic and clinical data were compared using Pearson’s chi-square test. The distribution of continuous variables was assessed for normality using the Shapiro–Wilk test and histogram test with a normal distribution curve. Logarithmic transformation was performed in data with non-normal distribution to achieve normal distribution. Normally distributed continuous samples were then compared using the independent sample t-test or one-way ANOVA followed by Tukey’s multiple comparisons test. For the associations that were significant in the univariate analysis, multivariable linear regression analysis was performed to adjust for age and gender etc. Pearson’s correlation was used to assess the correlation between the clinical ophthalmic parameters and the adhesion molecules. For the data from in vitro and animal studies, GraphPad Prism (Version 9.4.1, GraphPad Software, San Diego, CA, USA) was used for statistical analysis. Unpaired Student’s t-test was used for two group data and one or two-way ANOVA with Dunnett's multiple-comparisons test or Turkey’s multiple comparisons test was used for three and more group data analysis, respectively. All data were presented as mean ± standard deviation (SD).* P* values < 0.05 were considered statistically significant.

## Results

### Demographic and clinical characterization of participants

There were no significant differences in age, gender distribution, BCVA, intraocular pressure, body mass index (BMI), history of hypertension, or history of cardiovascular and cerebrovascular disease between controls and nAMD patients (Table 1).

**Table 1:** Demographic and clinical characteristics of nAMD patients and controls (Mean ± SD)

Varibles	All	Controls	nAMD	*P* value
	*n* = 72	*n* = 24	*n* = 48	
Age (y)	69.90 ± 7.71	70.17 ± 5.58	69.77 ± 8.63	0.815^a^
Female, n (%)	31 (43.1%)	14 (58.3%)	17 (35.4%)	0.064^b^
BCVA (LogMar)	0.75 ± 0.49	0.63 ± 0.57	0.81 ± 0.44	0.139^a^
Intraocular pressure (mmHg)	13.74 ± 2.34	13.46 ± 2.24	13.88 ± 2.41	0.481^a^
Body Mass Index (kg/m^2^)	23.90 ± 3.03	24.13 ± 2.48	23.79 ± 3.29	0.665^a^
History of hypertension, n (%)	33 (45.8%)	10 (41.7%)	23 (47.9%)	0.616^b^
History of cardiovascular disease, n (%)	10 (13.9%)	2 (8.3%)	8 (16.7%)	0.547^b^
History of cerebrovascular disease, n (%)	6 (8.3%)	1 (4.2%)	5 (10.4%)	0.651^b^

^a^Independent sample t-test

^b^Pearson’s chi-square test

Thirty-one out of 48 nAMD patients received anti-VEGF intravitreal injection before aqueous humour collection. The average duration between the last anti-VEGF injection and aqueous humour collection was 80.23 ± 116.03 days (interquartile range: 31 – 46 days). The average number of anti-VEGF injections prior to aqueous humour collection was 1.4 ± 1.4 (interquartile range 0–2). There was no correlation between the number of anti-VEGF injections and the intraocular levels of any of the adhesion molecules (ICAM-1: *r* = 0.243, *P* = 0.096; VCAM-1: *r* = 0.097, *P* = 0.513; CD44: *r* =  − 0.044, *P* = 0.765; CD62L: *r* =  − 0.055, *P* = 0.710; CD62P: *r* =  − 0.086, *P* = 0.561. Pearson's correlation analysis).

### Aqueous levels of adhesion molecule in nAMD patients with and without macular fibrosis

Macular fibrosis was present in 24 of the nAMD patients, including 12 in CNV and 12 in PCV patients. Except for gender, there was no significant difference in demographic and clinical characterizations such as age, BMI and history of hypertension, etc. between fibrosis present, fibrosis absent and the controls group (Supplementary Table S3). The adhesion molecules VCAM1, ICAM1, and CD44 were detected in all samples. CD62L was below the detection limit in seven samples from the control group. CD62P was not detected in three samples, including one from the fibrotic group and two from the non-fibrotic nAMD group. When nAMD patients (regardless of CNV or PCV) with macular fibrosis and those without were analyzed separately, both groups had significantly higher levels of ICAM-1, CD44 and CD62L compared to controls (Table 2). Interestingly, the aqueous level of VCAM-1 was significantly higher in nAMD patients with macular fibrosis but not in patients without macular fibrosis, compared to controls (Table 2). We also noted that patients with macular fibrosis generally had the highest levels of ICAM-1, VCAM-1, CD44 and CD62L among the three groups. The level of CD62P was comparable between control and different subtypes of nAMD patients (Table 2).

**Table 2:** The aqueous levels of ICAM-1, VCAM-1, CD44, CD62L and CD62P in nAMD patients with and without macular fibrosis (Mean ± SD)

Variables	Controls	Fibrosis absent	Fibrosis present	*P* value	*P* value	*P* value	*P* value	*P* value
				Fibrosis absent	Fibrosis absent vs	bonferroni	Fibrosis absent vs	adjust for
(Lg pg/ml)	*n* = 24	*n* = 24	*n* = 24	vs present^a^	present vs Con^b^	post hoc tests	present vs Con||	Gender||
ICAM-1	2.78 ± 0.30	3.15 ± 0.27	3.23 ± 0.34	0.413	< 0.001	< 0.001^c^	< 0.001	< 0.001^c^
						< 0.001^d^		< 0.001^d^
VCAM-1	3.79 ± 0.27	3.97 ± 0.44	4.21 ± 0.45	0.064	0.002	0.358^c^	0.001	0.097^c^
						0.001^d^		0.001^d^
CD44	2.17 ± 0.21	2.46 ± 0.09	2.50 ± 0.32	0.560	< 0.001	< 0.001^c^	< 0.001	< 0.001^c^
						< 0.001^d^		0.001^d^
CD62L	3.16 ± 0.28	3.66 ± 0.08	3.72 ± 0.19	0.188	< 0.001	< 0.001^c^	< 0.001	< 0.001^c^
						< 0.001^d^		< 0.001^d^
CD62P	2.08 ± 0.26	2.03 ± 0.36	2.10 ± 0.35	0.451	0.699	/	0.918	/

The data were log-transformed to obtain normal distribution

^a^Independent samples t-test

^b^One-way ANOVA

^c^Controls vs Fibrosis absent

^d^Controls vs fibrosis present

||Adjusted for gender with multivariable linear regression analysis

**Fig. 1:**
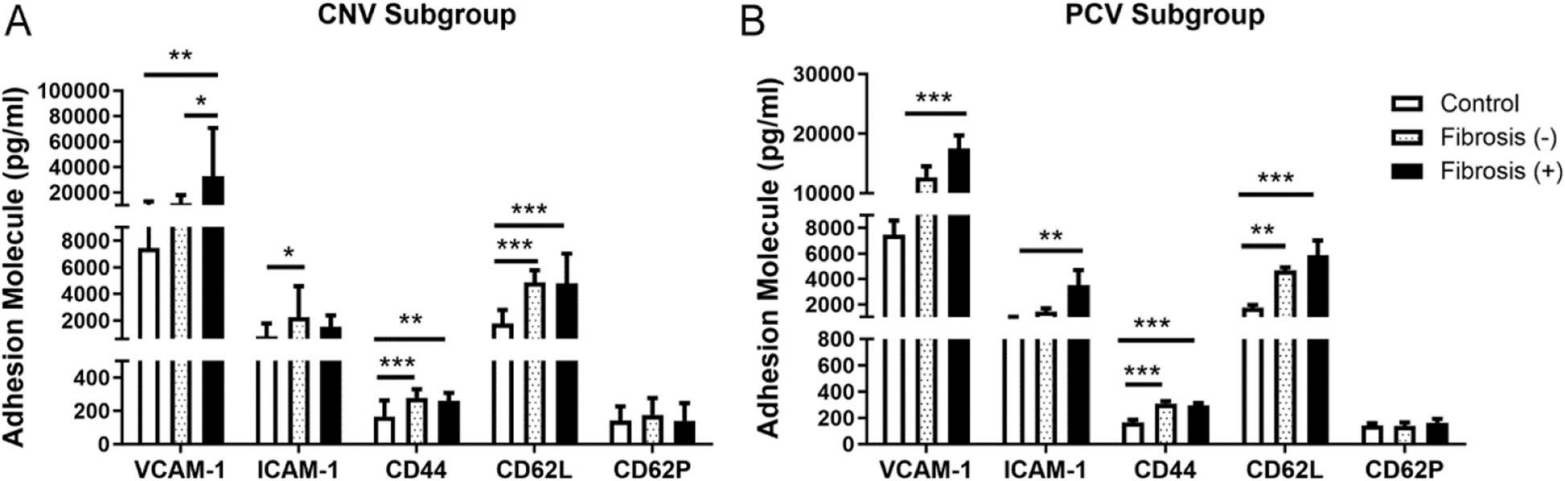
The levels of adhesion molecules in the aqueous humor in CNV and PCV subgroups with or without macular fibrosis. **A** The aqueous levels of ICAM-1, VCAM-1, CD44, CD62L and CD62P in CNV subgroup with and without macular fibrosis; **B** The aqueous levels of the same five adhesion molecules in the PCV subgroups with or without macular fibrosis. Mean ± SD (*P < 0.05; **P < 0.01; ***P < 0.001; *n* = 12 ~ 24/group). One-way ANOVA followed by Tukey’s multiple comparison’s test

We further investigated macular fibrosis presence vs absence in CNV and PCV subgroups. Participates in the PCV group was younger than those in control and CNV groups, whereas, participants in the CNV group had lower BMI (Supplementary Table S4). In CNV, the aqueous level of VCAM-1 was significantly higher in patients with macular fibrosis compared to those without (Fig. 1A). There were no significant differences in the levels of CD44 and CD62L between the two groups, although they were all significantly higher than those in controls (Fig. 1A). Surprisingly, the level of ICAM-1 was only significantly higher in patients without fibrosis (Fig. 1A). The levels of CD62P were comparable in the three groups.

In PCV, patients with fibrosis but not those without, had significantly higher levels of ICAM-1 and VCAM-1 compared to controls (Fig. 1B). The levels of CD44 and CD62L did not differ between fibrosis (-) and fibrosis ( +) patients, and both were significantly higher than controls (Fig. 1B). The levels of CD62P were comparable in the three groups (Fig. 1B).

### Correlation between intraocular levels of adhesion molecules and clinical presentations in nAMD patients

**Fig. 2:**
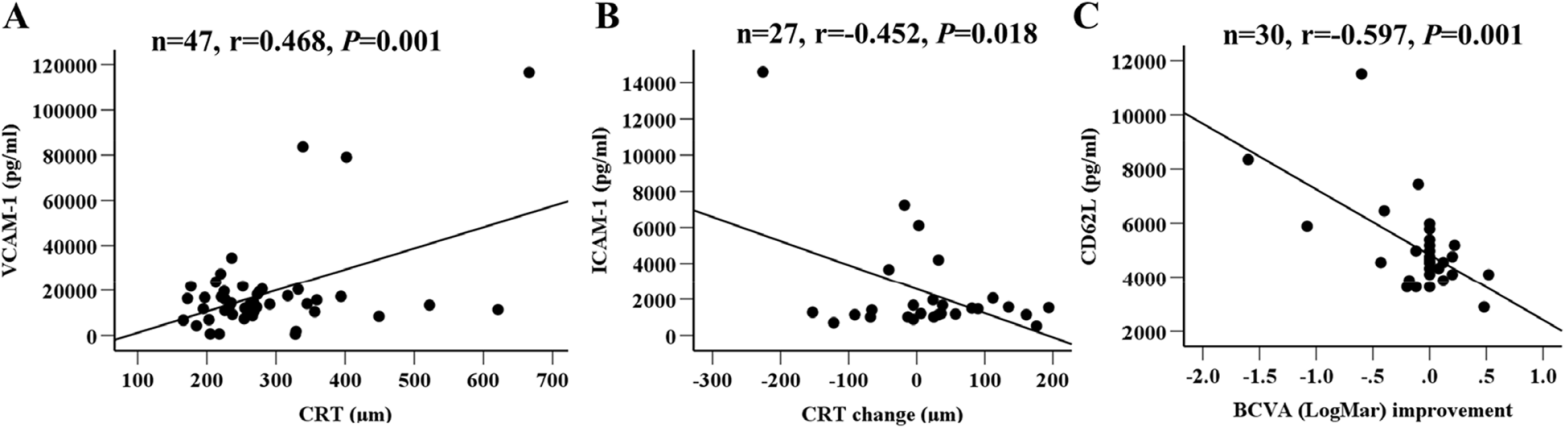
Correlations between the aqueous levels of adhesion molecules and clinical presentations. **A** Correlation between intraocular level of VCAM-1 and the CRT. **B** Correlation between intraocular levels of ICAM-1 and CRT change. **C** Correlation between intraocular level of CD62L and BCVA (LogMar) improvement. *n* = sample size, Pearson’s correlation analysis. BCVA (LogMar):best corrected visual acuity expressed as logMAR; CRT: central retinal thickness

Pearson correlation analysis showed that the level of VCAM-1 positively correlated with CRT (Fig. 2A), whereas the level of ICAM-1 negatively correlated with CRT change following anti-VEGF injection (Fig. 2B). The level of CD62L negatively correlated with BCVA improvement (Fig. 2C). We did not detect any correlation between any of the adhesion molecules and the age, BMI, and BCVA at the time of sample collection (Supplementary Table S5).

Taken together, our results suggest that nAMD patients had significantly higher intraocular levels of VCAM-1, ICAM-1, CD44 and CD62L and that higher level of VCAM-1 is related to macular fibrosis and increased central retinal thickness (a sign of macular oedema).

### The expression of VCAM-1 in subretinal fibrotic lesion

**Fig. 3:**
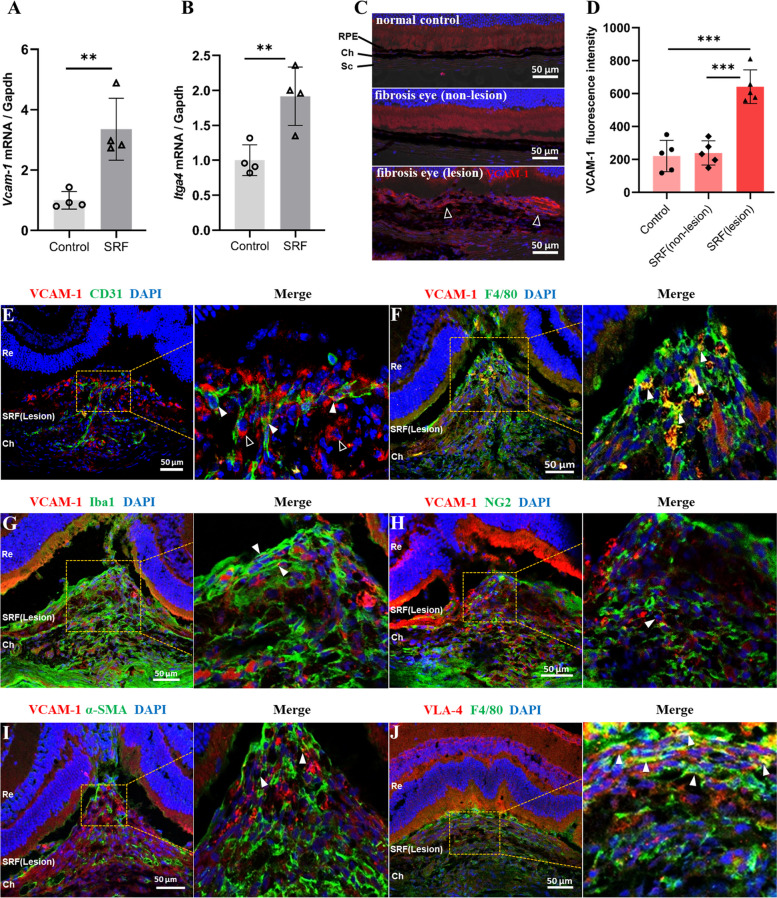
VCAM-1 expression in mouse eyes with/without subretinal fibrosis (SRF). **A**, **B** *Vcam-1* and *Itga4* mRNA expressions in RPE-choroid from control and subretinal fibrosis mouse eyes, *n* = 4 eyes, **P < 0.01, unpaired t test. **C** Representative confocal images showing VCAM-1 (red) expression in the out-retina layer, RPE/choroid/sclera of eyes from normal and subretinal fibrosis (5 days after the second laser) eyes. **D** Dot/bar figure showing mean fluorescence intensity of VCAM-1 in the choroid of normal eyes, non-lesion site and fibrotic lesions of fibrosis eyes. Mean ± SD, *n* = 5 eyes. ***P < 0.001, One-way ANOVA followed by Tukey’s multiple comparison. **E-I** Representative confocal images from subretinal fibrosis samples (5 days after the second laser) co-stained for VCAM-1^+^ (red) and CD31^+^ (green) (**E**) or F4/80 (**F**), or Iba-1 (**G**), or NG2 (**H**), or α-SMA (**I**). High magnification view of the yellow rectangle area is shown in the right. (**J**) Representative confocal images showing VLA-4 (red) expression in F4/80+ (green) macrophages in subretinal fibrosis. Hollow arrowheads in (**E**) indicate VCAM-1^+^CD31^−^ cells in the fibrotic lesions. White arrowheads indicate cells that co-express VCAM-1^+^ and other markers. SRF = Subretinal fibrosis, Re = Retina, Ch = Choroid, Sc = Scleral

To further understand the role of VCAM-1 in the pathogenesis of macular fibrosis, we investigated expression of *Vcam1* and its receptor *Itga4* genes in the mouse model of subretinal fibrosis. Our result shows that both *Vcam1* and *Itga4* were significantly higher in RPE/choroid with subretinal fibrosis than that in controls (Fig. 3A, B). Increased VCAM-1 expression was further confirmed at the protein level by confocal microscopy (Fig. 3C, D). VCAM-1^+^ cells were detected in the subretinal fibrosis lesion (hollow arrowheads, Fig. 3C), but only weakly present in non-lesion area of fibrotic eyes and normal eyes. The expression levels of VCAM-1 in the choroid of normal mice and the non-lesion sites of fibrosis eyes were comparable (Fig. 3D). Dual staining of VCAM-1 and CD31 showed that many CD31^+^ vascular endothelia cells were positive for VCAM-1 inside the fibrotic lesion (arrowheads, Fig. 3E), although there were also many CD31^−^VCAM-1^+^ cells (hollow arrowheads, Fig. 3E). To understand the nature of CD31^−^VCAM-1^+^ cells, we co-stained VCAM-1 with other cell markers, including macrophages (F4/80 or IBA-1), pericytes (NG2), and myofibroblasts (α-SMA). Our results show that many F4/80^+^ macrophages and Iba1^+^ myeloid cells were positive for VCAM-1 inside the fibrotic lesion (arrowheads, Fig. 3F, G). Few NG2^+^ (Fig. 3H) or α-SMA^+^ (Fig. 3I) cells were positive for VCAM-1, but the majority of NG2^+^ or α-SMA^+^ cells were negative for VCAM-1 (Fig. 3H, I). The expression of VLA-4 (the primary receptor for VCAM-1) was detected predominately in F4/80^+^ macrophages (arrowheads in zoomed image of Fig. 3J). Our results suggest that the main cellular sources of VCAM-1 in our model of subretinal fibrosis are vascular endothelial cells and infiltrating macrophages and macrophages are also the key target of VCAM-1.

### The effect of blocking VCAM-1 or its receptor VLA-4 on subretinal fibrosis

**Fig. 4:**
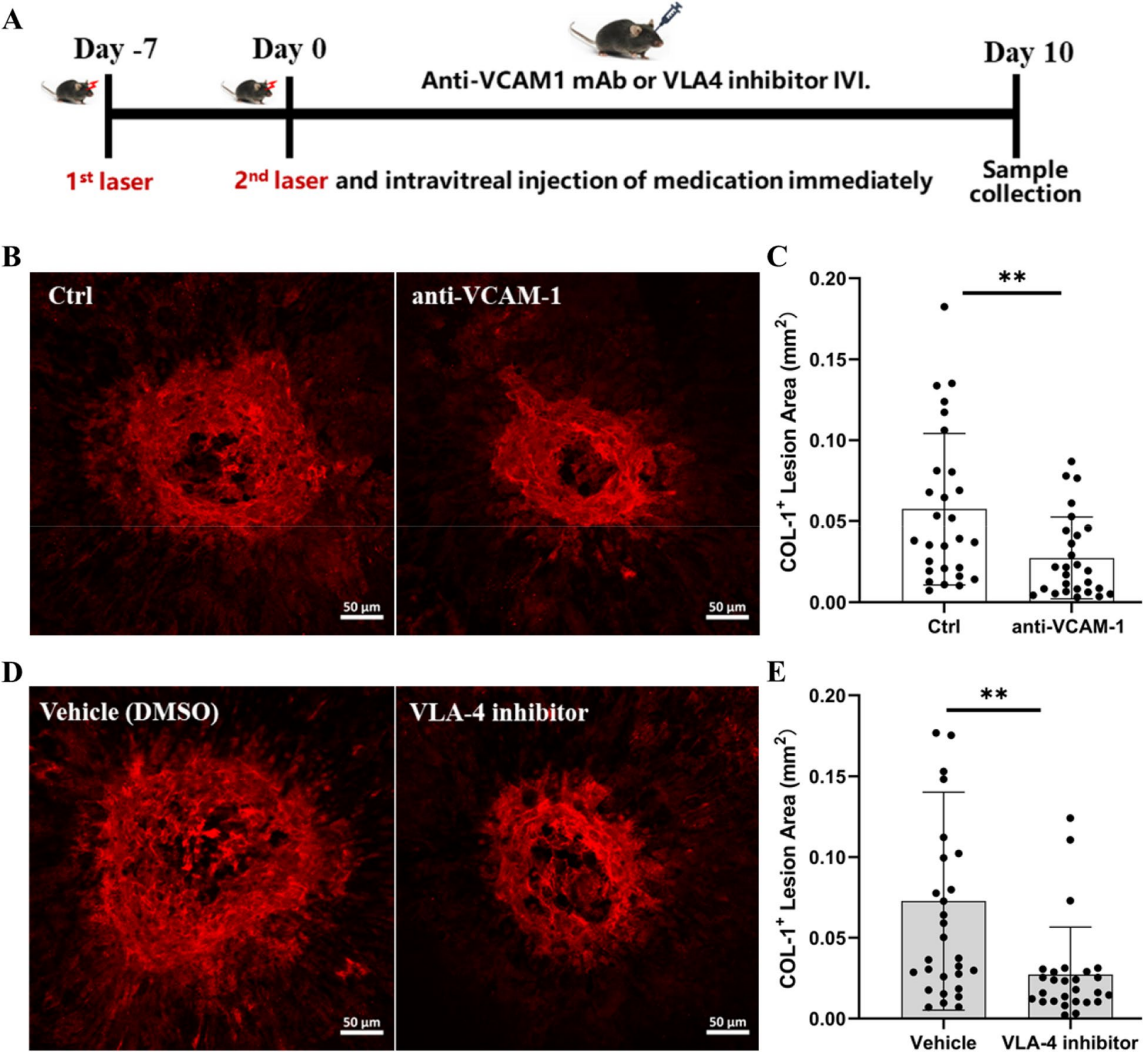
The effect of anti-VCAM-1 antibody and VLA4 inhibitor on subretinal fibrosis. **A** Schematic diagram showing experimental design. Mice were treated with intravitreal injection (IVI) of anti-VCAM-1 mAb, VLA-4 inhibitor or vehicle immediately after the second laser. Eyes were collected on day 10 for immunofluorescence staining. **B** Representative images of RPE/choroid flatmounts stained for collagen-1 (COL-1); **C** quantitative analysis of COL-1^+^ lesion areas. **D** Representative images of COL-1 staining of RPE/choroid flatmounts; **E** quantification of COL-1^+^ lesion areas. Graphs represent mean ± SD, *n* = 28 lesions per group from 7 eyes, **P < 0.01, unpaired t test

To understand the pathogenic role of VCAM-1 and its receptor VLA-4 in macular fibrosis, we used a VCAM-1 blocking antibody (BE0027) and a VLA-4 inhibitor (BIO5192) in our two-stage laser-induced mouse model of subretinal fibrosis (Fig. 4A). Our results show that both anti-VCAM-1 antibody (Fig. 4B, C) and VLA-4 inhibitor (Fig. 4D, E) significantly reduced the size of collagen-1^+^ fibrotic lesions.

**Fig. 5:**
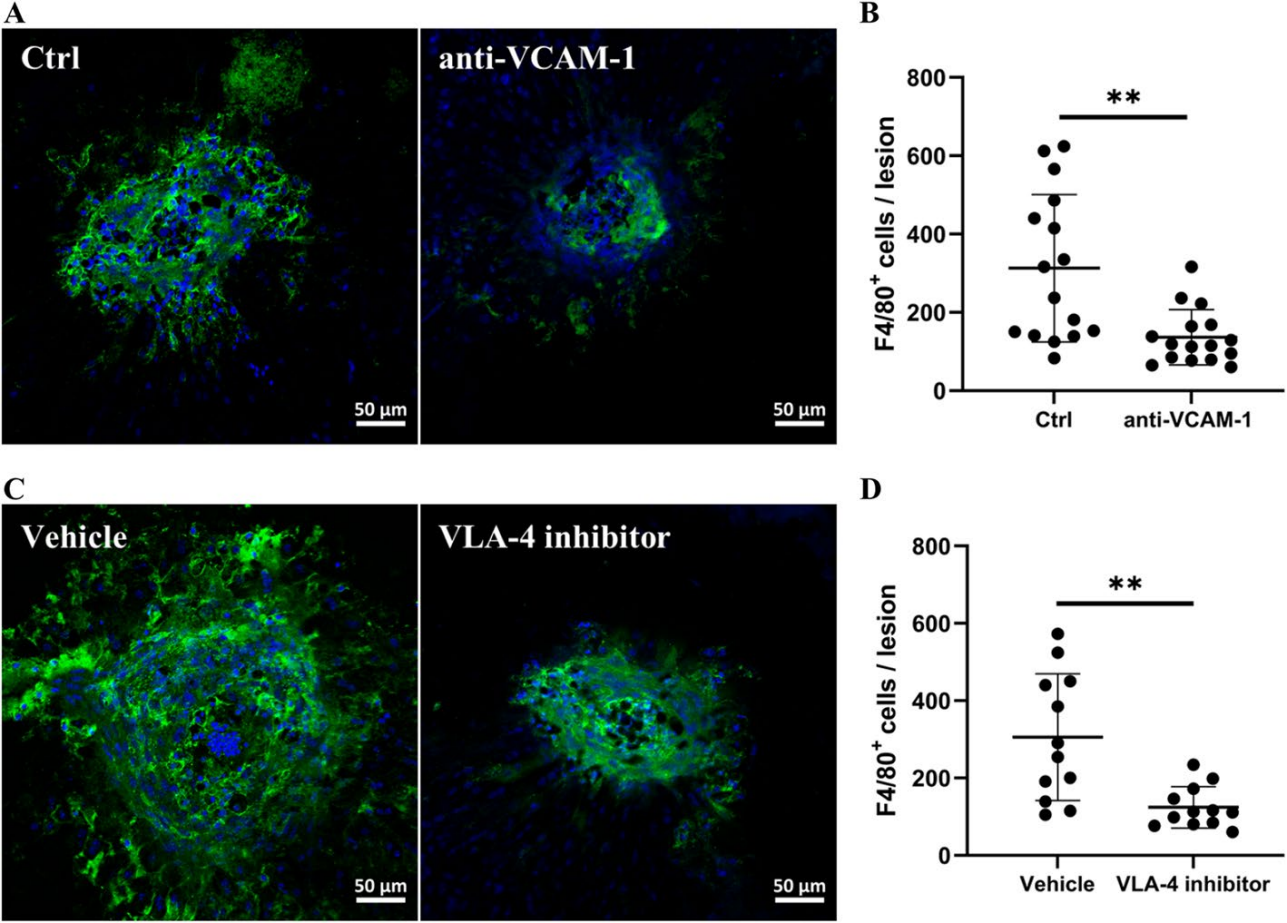
The effect of anti-VCAM-1 antibody or VLA-4 inhibitor on macrophage infiltration in the mouse model of subretinal fibrosis. **A**, **C** Representative images of RPE/choroid flatmounts from different groups stained for F4/80 (green) and DAPI. **B**, **D** Quantification of the F4/80^+^ cells in different groups. Mean ± SD, *n* = 12–16 lesions from 3–4 eyes, **P < 0.01, unpaired t test

Infiltrating macrophages critically contribute to subretinal fibrosis [19, 20, 33]. The VCAM-1/VLA-4 axis is known to play a critical role in leukocyte trafficking during inflammation [34]. We found that the number of infiltrating F4/80^+^ macrophages was significantly reduced in anti-VCAM-1 neutralizing antibody-treated (Fig. 5A, B) and VLA-4 inhibitor-treated eyes (Fig. 5C, D). This result suggests that blocking VCAM-1 or its receptor, VLA-4, reduced macrophage infiltration in our mouse model of subretinal fibrosis.

### The effect of VCAM-1 on macrophage migration

**Fig. 6:**
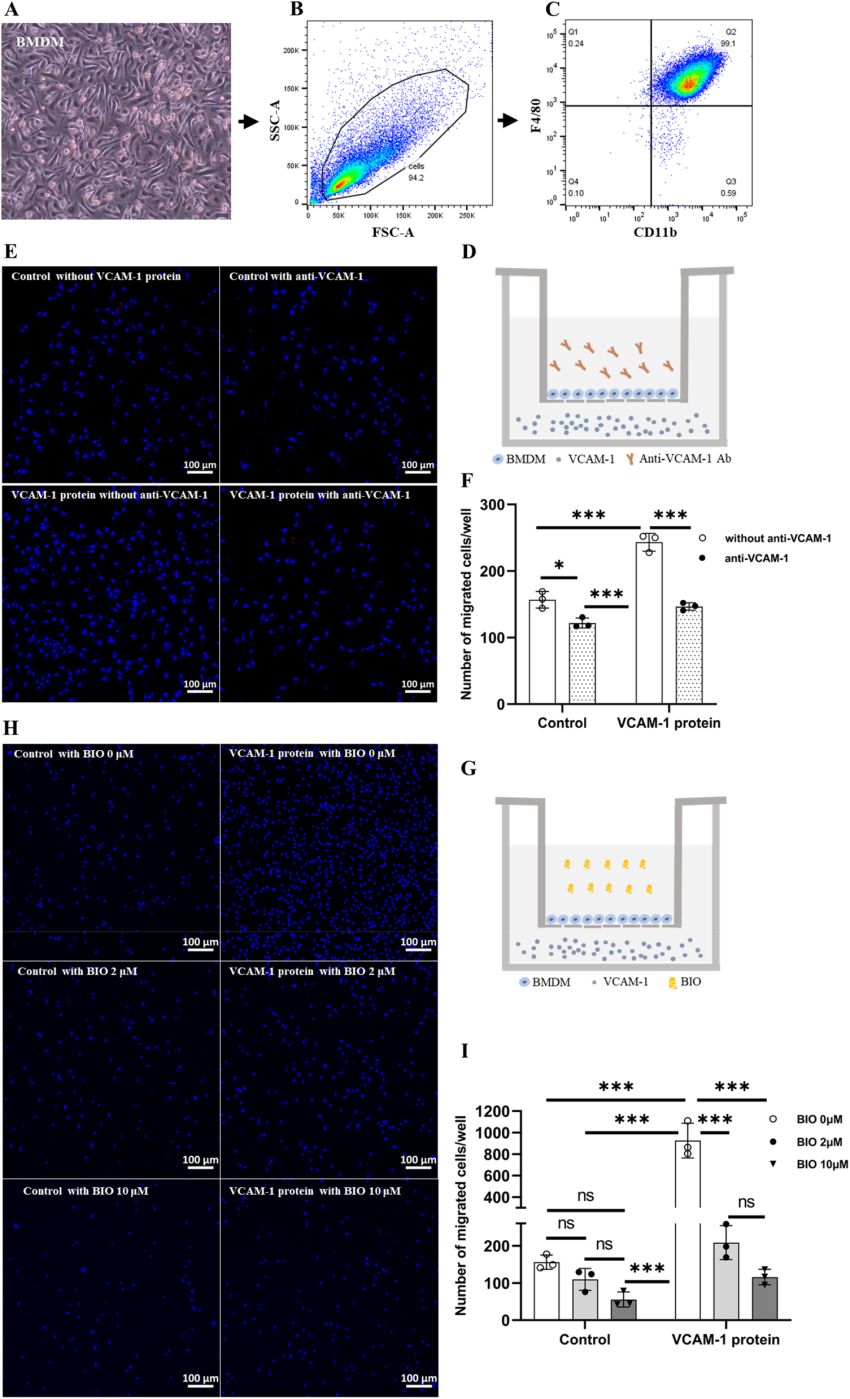
The effect of VCAM-1 on BMDM migration. **A** Phase-contrast image showing the morphology of BMDM cultures. **B**, **C** Flow cytometry analysis of BMDMs. Live cells were gated on FSC-A and SSC-A to remove debris (**B**) and before plotting their CD11b and F4/80 expression (**C**). **D** Diagram of the migration assay using the Boyden Chamber system. BMDMs were added into a transwell insert with a porous membrane. The recombinant VCAM-1 protein (25 μg/ml) was added into the bottom chamber. The anti-VCAM-1 Ab (20 µg/ml) was added to the upper chamber. **E** Representative images of migrated macrophages on the bottom side of the inserts stained with DAPI from each treatment group. **F** Bar graph showing the number of migrated cells in different groups. *P < 0.05, *n* = 3. Mean ± SD. Two-way ANOVA followed by Turkey’s multiple comparisons test. **G** Experimental design to detect the effect of blocking VLA-4 using BIO5192 in VCAM-1-induced macrophage migration. **H** Representative images of migrated macrophages on the bottom side of the inserts stained with DAPI from each treatment group. **I** Bar graph showing the number of migrated cells in different groups. *P < 0.05, *n* = 3. Mean ± SD. Two-way ANOVA followed by Turkey’s multiple comparisons test

To further understand the mechanism of VCAM-1/VLA-4 blockade mediated protection in subretinal fibrosis, we investigated the effect of recombinant VCAM-1 on macrophage migration using the Boyden Chamber system. The phenotype of BMDMs was confirmed by flow cytometry (> 95% of the cells were CD11b^+^ F4/80^+^ (Fig. 6A-C).

The cells were placed inside the upper chamber with/without neutralizing anti-VCAM-1 antibody (20 μg/ml) or VLA-4 inhibitor (BIO5192). Mouse recombinant VCAM-1 (25 µg/ml) was added to the bottom chamber (Fig. 6D, G). After 10 h incubation, cells in the outside of the inserts were counted. Recombinant VCAM-1-treated group had significantly higher number of cells migrated to the bottom layer of the inserts and this effect was blocked by anti-VCAM-1 antibody (Fig. 6E, F) and VLA-4 inhibitor BIO5192 (2, 10 µM) (Fig. 6H, I). Interesting, blocking VCAM-1 or VLA-4 also significantly reduced macrophage migration in the control group (i.e., without VCAM-1 stimulation). The results suggest that the VCAM-1/VLA-4 axis is also involved in macrophage migration under normal conditions (i.e., spontaneous migration).

### The effect of VCAM-1 on macrophage gene expression

**Fig. 7:**
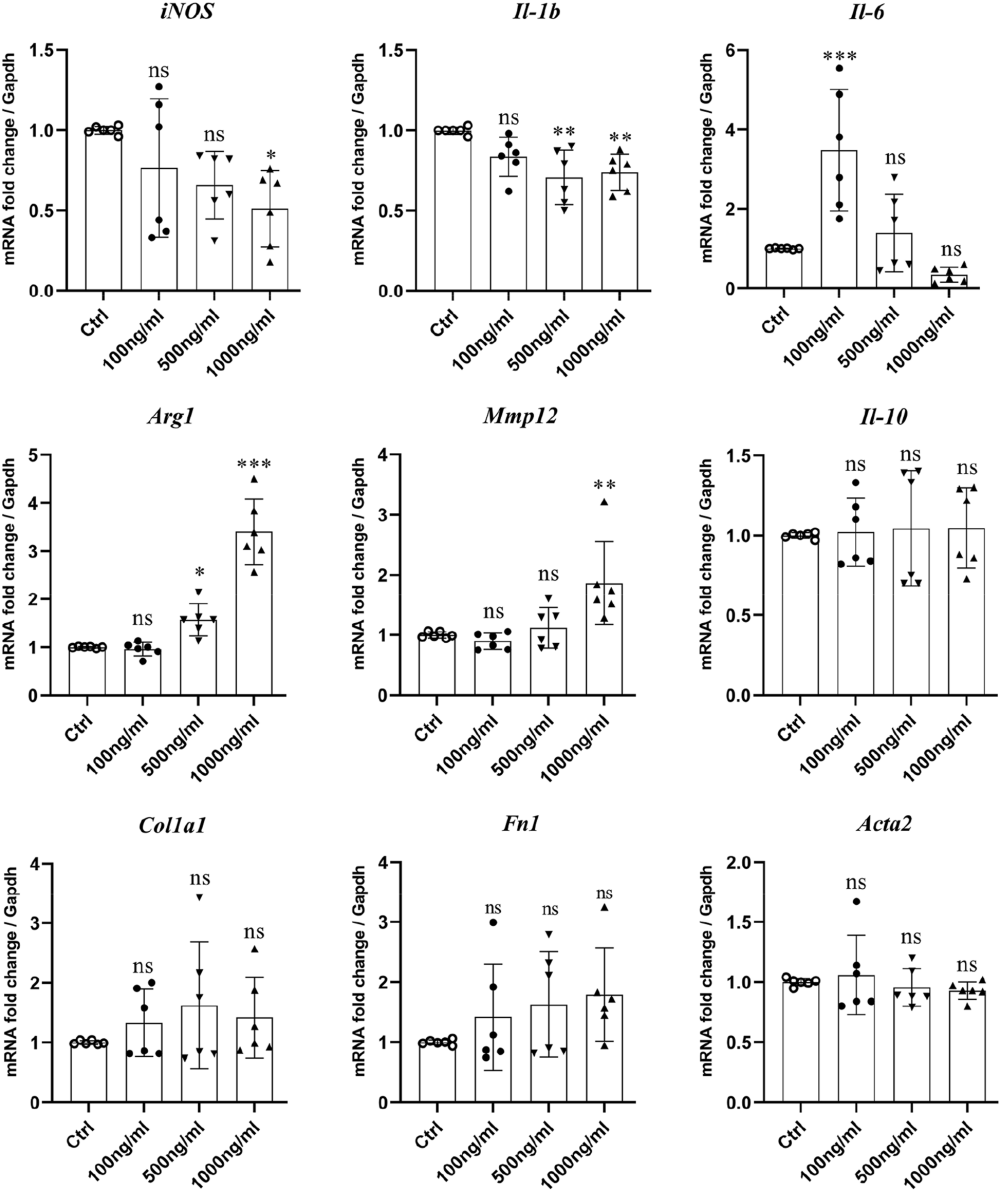
The effect of VCAM-1 on BMDM activation and macrophage-to-myofibroblast transition. BMDM were treated with or without different concentrations of recombinant murine VCAM-1 protein (100, 500, and 1000 ng/ml) for 24 h. The expression of *iNOS, Il1b, Il6, Arg1, Mmp12, Il10, Col1a1*, *Fn1* and *Acta2* was examined by RT-qPCR. Mean ± SD, *n* = 6, *P < 0.05, **P < 0.01, ***P < 0.001. One-way ANOVA followed by Dunnett's test

To investigate the effect of VCAM-1 on macrophage activation/polarization, we treated BMDMs with different concentrations of VCAM-1 and examined their inflammatory and pro-fibrotic gene expression. The results show that VCAM-1 treatment significantly reduced the expression of *iNOS and Il1b,* (Fig. 7) but increased the expression of *Il6, Arg-1and Mmp12* (Fig. 7). We previously reported that macrophage-to-myofibroblast transition (MMT) is involved in macular fibrosis [19, 20]. We did not detect any changes in the expression of *Col1a1*, *Fn1* and *Acta2* in VCAM-1 treated BMDM (Fig. 7).

## Discussion

In this study, we found that the intraocular levels of CD62L, CD44, ICAM-1, and VCAM-1 were significantly higher in nAMD patients compared to age-matched controls. Importantly, we found that the intraocular level of VCAM-1 was significantly higher in patients with macular fibrosis than those without. In vivo treatment with VCAM-1 neutralizing antibody or its receptor VLA-4 inhibitor reduced macrophage infiltration and suppressed subretinal fibrosis. We further found that VCAM-1 could induce macrophage migration and alter inflammatory gene expression in macrophages. Our results suggest that VCAM-1 critically contributes to macular fibrosis through modulating macrophage functions.

Adhesion molecules are expressed predominately by immune cells and vascular endothelial cells. Their expressions are upregulated during inflammation to facilitate the recruitment and migration of immune cells. The adhesion molecules can be released upon cell activation or death. The soluble form of these molecules can further enhance immune cell activation and facilitate extravasation during inflammation. ICAM-1 and VCAM-1 are expressed predominantly by endothelial cells (although low levels of ICAM-1 are also expressed in some immune cells) and play a critical role in leukocyte trafficking during inflammation [34, 35]. Previous studies have shown that VCAM-1 expression in endothelial cells is increased after TNFα treatment [36] and the expression of VCAM-1 is strongly associated with increased intimal leukocyte accumulation [37]. CD44 is expressed on the surface of immune cells, epithelial cells, and mesenchymal cells. The expression of CD44 in immune cells is increased upon activation and a higher level of CD44 is required for their recruitment to the site of inflammation. We previously reported that CD44 is critically involved in leukocyte trafficking across the BRB in the inflamed retina [21]. CD62L (L-selectin) is expressed on lymphocytes (T and B cells) and myeloid cells such as neutrophils and is shed from the cell surface after cell activation. During inflammation, the cell surface adhesion molecules can also be cleaved by enzymes such as ADAM17 and MMP9 [38, 39]. An abnormal level of MMP9 has been reported in nAMD patients [40, 41]. The increased intraocular levels of ICAM-1 and VCAM-1 may suggest higher levels of endothelial cell activation, whereas higher levels of CD44 and CD62L indicate excessive intraocular immune cell activation in nAMD.

We found the intraocular level of VCAM-1 was significantly higher in nAMD patients with macular fibrosis and was positively correlated with central retinal (macular) thickness. Since increased macular thickness (macular oedema) in nAMD is due to the leakage from the diseased blood vessels, the positive correlation highlights the excessive vascular endothelial activation and dysfunction in nAMD with subretinal macular fibrosis. We found that blocking VCAM-1 or its receptor VLA-4 suppressed fibrosis in a mouse model CNV-mediated macular fibrosis. Fibrosis in nAMD is the conversion of new blood vessels into fibro-vascular membrane through myofibroblast activation [42]. Fibroblasts do not exist in the retina/macula, but multiple pathways are known to be involved in the recruitment, differentiation, and activation of myofibroblasts. Potential sources of myofibroblasts in nAMD include circulating fibrocytes [33], RPE cells (through epithelial-mesenchymal transition, EMT) [43], macrophages (through macrophage-to-myofibroblast transition, MMT) [19], endothelial cells, pericytes and perivascular mesenchymal stem cells [44, 45]. A previous study has shown that VCAM-1 is a TGF-β1 responsive mediator that partakes in fibroblast proliferation in idiopathic pulmonary fibrosis [46]. VCAM-1 is also reported to be involved in smooth muscle cell activation and migration [47]. Blocking VCAM-1/VLA-4 pathway may suppress macular fibrosis through multiple mechanisms including the recruitment of fibrocytes and macrophages and modulating the function of tissue and immune cells (see below).

The VCAM-1 receptor, the integrin α4β1 (VLA-4) is expressed on monocytes and macrophages. We found that soluble VCAM-1 can promote macrophage migration, which is in line with a previous report by Takahashi et al. [48], whereby the authors showed that soluble VCAM-1 could promote macrophage infiltration and retention in tumor microenvironment that conferred resistance to chemotherapy [48]. We previously reported that macrophages could contribute to macular fibrosis through MMT [20] and macrophage elastase (MMP12) is critically involved in this process [20]. Although we did not detect any effect of VCAM-1 on macrophage MMT in the current study, we did find that VCAM-1 could reduce *iNOS, Il1b* expression and increase *Il6, Mmp12,* and *Arg1* expression in macrophages. Both IL-6 [49] and MMP12 can promote fibrosis. Our results suggest that VCAM-1 may contribute to macular fibrosis by modulating macrophage differentiation into an alternatively activated profibrotic phenotype. Further studies will be needed to understand whether VCAM-1 is also involved in fibrocyte recruitment and the process of EMT from RPE or vascular endothelial cells in macular fibrosis.

## Conclusions

In this study, we identified higher intraocular level of VCAM-1 as an important contributor of CNV-mediated macular fibrosis in nAMD. nAMD patients with macular fibrosis had a significantly higher intraocular level of VCAM-1 than those without macular fibrosis. Blocking VCAM-1 or its receptor VLA-4 significantly suppressed fibrosis in a mouse model of CNV-mediated macular fibrosis. Mechanistically, VCAM-1 can promote macrophage migration and modulate macrophage function towards an alternatively activated pro-fibrotic phenotype. The intraocular VCAM-1 may be targeted for the prevention or treatment of CNV-mediated macular fibrosis.

### Supplementary Information

**Additional file 1: Supplementary Table S1.** The primary and secondary antibodies used in immunofluorescence staining. **Supplementary Table S2.** Primer sequences of mouse for real-time qPCR. **Supplementary Table S3.** The clinical baseline data of fibrosis absent, fibrosis present and the controls group. **Supplementary Table S4.** The clinical baseline data of CNV, PCV and the controls group. **Supplementary Table S5.** Correlation between intraocular levels of adhesion molecules and clinical presentations in nAMD patients. **Supplementary Figure S1.** Immunofluorescence staining confirmed the expression of VCAM-1 and VLA-4 in F4/80^+^ macrophage.

## Data Availability

The datasets generated and analysed during the current study are available from the corresponding author on reasonable request.

## Abbreviations

AMD: Age-related macular degeneration
CNV: Choroidal neovascularization
MMT: Macrophage-to-myofibroblast transition
nAMD: Neovascular age-related macular degeneration
PCV: Polypoidal choroidal vasculopathy
SRF: Subretinal fibrosis
