# Supplementary material for: Vascular Cell Adhesion Molecule-1 (VCAM-1) contributes to macular fibrosis in neovascular age-related macular degeneration through modulating macrophage functions
Source: Immun Ageing. 2023 Nov 20;20:65. doi: 10.1186/s12979-023-00389-x (PMC10659061; doi:10.1186/s12979-023-00389-x)
Supplement: Supplementary file 1 — Additional file 1: Supplementary Table S1. The primary and secondary antibodies used in immunofluorescence staining. Supplementary Table S2. Primer sequences of mouse for real-time qPCR. Supplementary Table S3. The clinical baseline data of fibrosis absent, fibrosis present and the controls group. Supplementary Table S4. The clinical baseline data of CNV, PCV and the controls group. Supplementary Table S5. Correlation between intraocular levels of adhesion molecules and clinical presentations in nAMD patients. Supplementary Figure S1. Immunofluorescence staining confirmed the expression of VCAM-1 and VLA-4 in F4/80+ macrophage. [file 12979_2023_389_MOESM1_ESM.docx]

**Vascular cell adhesion molecule-1 (VCAM-1) contributes to macular fibrosis in neovascular age-related macular degeneration through modulating macrophage functions**

Wen Deng^1,2^, Caijiao Yi^2^, Wei Pan^2^, Jian Liu^2^, Jinyan Qi^1,2^, Juan Chen^3^, Zengchao Zhou^3^, Yiqin Duan^3^, Xiangyan Ning^3^, Jun Li^3^, Changhua Ye^1,3^, Zhongping Chen^1,3^ and Heping Xu^1,2,4*^

*Supplementary Tables and Figures accompany this paper:*

**Table S1.** The primary and secondary antibodies used in immunofluorescence staining.

| **Antibodies** | **Dilution** | **Sources** | **Catalog numbers** |
| --- | --- | --- | --- |
| **Primary antibodies** | | | |
| Mouse monoclonal anti-Iba1 | 1:200 | Abcam | ab283319 |
| Mouse monoclonal anti-αSMA | 1:200 | Abcam | ab7817 |
| Mouse monoclonal anti-NG2 | 1:100 | Santa Cruz | sc-53389 |
| Rat monoclonal anti-F4/80 | 1:200 | Abcam, | ab6640 |
| Rat monoclonal anti-CD31 | 1:200 | Invitrogen | MA1-40074 |
| Rabbit monoclonal anti-VCAM-1 | 1:200 | Abcam | ab134047 |
| Rabbit polyclonal anti-Collagen I | 1:200 | Abcam | ab34710 |
| Rabbit monoclonal anti-VLA-4 | 1:100 | Proteintech | 19676-1-AP |
| **Secondary antibodies** | | | |
| Alexa Fluor® 488 goat anti-mouse IgG | 1:400 | Invitrogen | A-11001 |
| Alexa Fluor® 488 goat anti-rat IgG | 1:400 | Invitrogen | A-11006 |
| Alexa Fluor® 594 goat anti-rabbit IgG | 1:400 | Invitrogen | A-32740 |

**Table S2.** Primer sequences of mouse genes for real-time qPCR

| **Primer gene** | **Forward sequence 5'-3'** | **Reverse sequence 5'-3'** | |
| --- | --- | --- | --- |
| *Vcam-1*  *Itga4* | TCAGGAAATGCCACCCTCAC  CACTCCAGCCGATCCTTCAG | CAGCACACGTCAGAACAACC  TCTTCATGCTCCCAACAGCA | |
| *Gapdh* | TGGCAAAGTGGAGATTGTTGC | AAGATGGTGATGGGCTTCCCG | |
| *iNOS* | GTTCTCAGCCCAACAATACAAGA | GTGGACGGGTCGATGTCAC | |
| *Il-1b* | GCAACTGTTCCTGAACTCAACT | ATCTTTTGGGGTCCGTCAACT | |
| *Il-6* | CTGCAAGAGACTTCCATCCAG | AGTGGTATAGACAGGTCTGTTGG | |
| *Il-10* | CTTACTGACTGGCATGAGGATCA | GCAGCTCTAGGAGCATGTGG | |
| *Arg1* | CTCCAAGCCAAAGTCCTTAGAG | GGAGCTGTCATTAGGGACATCA | |
| *Mmp12* | CCTGCTTACCCCAAGCTGAT | ATGTTTTGGTGACACGACGG | |
| *Col1a1* | GTGGCGGTTATGACTTCAGC | GGCTGCGGATGTTCTCAATC | |
| *Fn1* | CCACTTCCCCTTCCTGTACA | ATCGTAGTTCTGGGTGGTGC |  |
| *Acta2* | TGGCACCACTCTTTCTATAACG | GGTCATTTTCTCCCGGTTGG |  |

| **Table S3.** The clinical baseline data of fibrosis absent, fibrosis present and the controls group (Mean ± SD) | | | | |
| --- | --- | --- | --- | --- |
| **Variables** | **Controls** | **Fibrosis absent** | **Fibrosis present** | ***P* value** |
|  |  |  |  | Fibrosis absent vs |
|  | n=24 | n=24 | n=24 | present vs Con |
| Age (y) | 70.17±5.58 | 70.29±9.58 | 69.25±7.74 | 0.881* |
| Female, n (%) | 14 (58.3%) | 14 (58.3%) | 3 (12.5%) | **0.001†** |
| BCVA (LogMar) | 0.63±0.57 | 0.61±0.34 | 1.01±0.44 | **0.005*** |
| Intraocular pressure (mmHg) | 13.46±2.24 | 13.71±2.96 | 14.05±1.74 | 0.694* |
| Body Mass Index（kg/m^2^） | 24.13±2.48 | 23.53±3.65 | 24.06±2.94 | 0.762* |
| History of hypertension, n (%) | 10 (41.7%) | 12 (50%) | 11 (45.8)% | 0.845† |
| * One-way ANOVA, † Pearson’s chi-square test, SD standard deviation, *P* < 0.05. | | | | |

| **Table S4.** The clinical baseline data of CNV, PCV and the controls group (Mean ± SD) | | | | |
| --- | --- | --- | --- | --- |
| **Variables** | **Controls** | **CNV** | **PCV** | ***P* value** |
|  |  |  |  | Controls vs |
|  | n=24 | n=24 | n=24 | CNV vs PCV |
| Age (y) | 70.17±5.58 | 72.75±7.54 | 66.79±8.77 | **0.025*** |
| Female, n (%) | 14 (58.3%) | 9 (37.5%) | 8 (33.3%) | 0.173† |
| BCVA (LogMar) | 0.63±0.57 | 0.81±0.49 | 0.81±0.38 | 0.338* |
| Intraocular pressure (mmHg) | 13.46±2.24 | 13.55±2.34 | 14.21±2.48 | 0.484* |
| Body Mass Index（BMI, kg/m^2^） | 24.13±2.48 | 22.93±3.25 | 24.66±3.16 | 0.126* |
| History of hypertension, n (%) | 10 (41.7%) | 13 (54.2%) | 10 (41.7%) | 0.604† |
| History of cardiovascular disease, n (%) | 2 (8.3%) | 4 (16.7%) | 4 (16.7%) | 0.756† |

* One-way ANOVA, † Pearson’s chi-square test, SD standard deviation, *P* < 0.05.

| **Table S5**. Correlation between intraocular levels of adhesion molecules and clinical presentations in nAMD patients. | | | | | | | | | | |
| --- | --- | --- | --- | --- | --- | --- | --- | --- | --- | --- |
| **Clinical data** | **ICAM-1** | | **VCAM-1** | | **CD44** | | **CD62L** | | **CD62P** | |
|  | r | *P* | r | *P* | r | *P* | r | *P* | r | *P* |
| Age (n=48) | 0.123 | 0.403 | 0.139 | 0.346 | 0.169 | 0.252 | 0.238 | 0.103 | 0.272 | 0.061 |
| BMI (n=48) | -0.058 | 0.694 | 0.184 | 0.209 | -0.044 | 0.766 | 0.017 | 0.909 | 0.168 | 0.254 |
| BCVA (n=48) | -0.004 | 0.981 | 0.209 | 0.155 | -0.039 | 0.791 | 0.156 | 0.290 | 0.001 | 0.994 |
| BCVA improvement (n=30) | -0.275 | 0.142 | -0.132 | 0.488 | -0.219 | 0.245 | -0.597 | **0.001** | -0.312 | 0.093 |
| CRT (n=47) | -0.158 | 0.288 | 0.468 | **0.001** | 0.065 | 0.663 | 0.051 | 0.733 | 0.018 | 0.902 |
| CRT change (n=27) | -0.452 | **0.018** | 0.009 | 0.965 | -0.102 | 0.612 | 0.115 | 0.568 | 0.026 | 0.898 |
| r: Pearson correlation coefficient; *P*: statistical test value; BMI: body mass index; BCVA: logMAR best corrected visual acuity; CRT: central retinal thickness. | | | | | | | | | | |

**
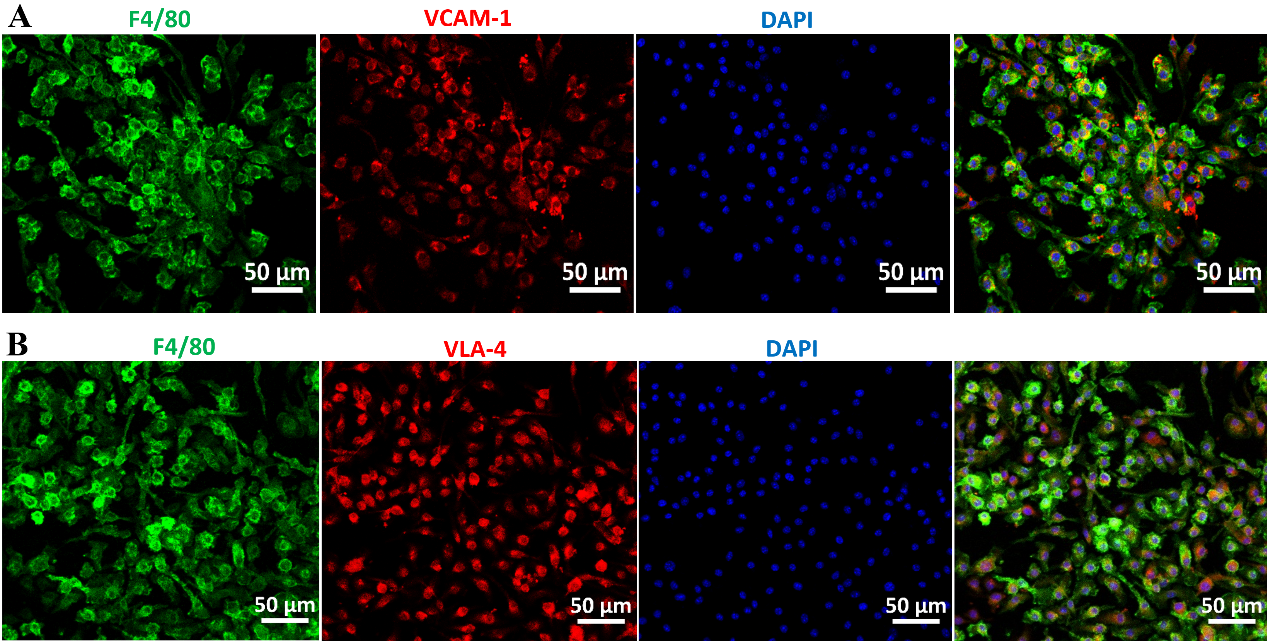
**

**Figure S1.** Immunofluorescence staining of VCAM-1 and VLA-4 in bone marrow derived macrophages (BMDMs). (A) Representative images showing the expression of F4/80 (green) and VCAM-1 (red) in BMDMs. (B) Representative images showing the expression of F4/80 (green) and VLA-4 (red) in BMDMs.
